# CRISPR/Cas9-Mediated Integration of Large Transgene into Pig *CEP112* Locus

**DOI:** 10.1534/g3.119.400810

**Published:** 2019-12-09

**Authors:** Guoling Li, Xianwei Zhang, Haoqiang Wang, Jianxin Mo, Cuili Zhong, Junsong Shi, Rong Zhou, Zicong Li, Huaqiang Yang, Zhenfang Wu, Dewu Liu

**Affiliations:** * National Engineering Research Center for Breeding Swine Industry, College of Animal Science, South China Agricultural University, Guangzhou 510642, China and; † Wens Foodstuff Group Co., Ltd, Yunfu 527400, China

**Keywords:** CRISPR/Cas9, *CEP112*, homology arm, genetically modified pig, safe harbor

## Abstract

Clustered regularly interspaced short palindromic repeats (CRISPR)-associated protein 9 (Cas9) is a precise genome manipulating tool that can produce targeted gene mutations in various cells and organisms. Although CRISPR/Cas9 can efficiently generate gene knockout, the gene knock-in (KI) efficiency mediated by homology-directed repair remains low, especially for large fragment integration. In this study, we established an efficient method for the CRISPR/Cas9-mediated integration of large transgene cassette, which carries salivary gland-expressed multiple digestion enzymes (≈ 20 kbp) in *CEP112* locus in pig fetal fibroblasts (PFFs). Our results showed that using an optimal homology donor with a short and a long arm yielded the best CRISPR/Cas9-mediated KI efficiency in *CEP112* locus, and the targeting efficiency in *CEP112* locus was higher than in *ROSA26* locus. The *CEP112* KI cell lines were used as nuclear donors for somatic cell nuclear transfer to create genetically modified pigs. We found that KI pig (705) successfully expressed three microbial enzymes (β-glucanase, xylanase, and phytase) in salivary gland. This finding suggested that the *CEP112* locus supports exogenous gene expression by a tissue-specific promoter. In summary, we successfully targeted *CEP112* locus in pigs by using our optimal homology arm system and established a modified pig model for foreign digestion enzyme expression in the saliva.

Genetically modified animals play an important role in agricultural and biomedical studies. Zinc-finger nucleases, transcription activator-like effector nucleases, and clustered regularly interspaced short palindromic repeats (CRISPR)-associated protein 9 (Cas9) system can enable the targeted generation of DNA double strand breaks (DSBs) with high accuracy and efficiency (Doudna and Charpentier 2014; Hsu *et al.* 2014). The induced DSBs trigger two major DNA repair systems, namely, non-homologous end joining (NHEJ) and homology-directed repair (HDR) (You *et al.* 2009). The NHEJ pathway results in gene knockout (KO) by generating randomly sized small insertions or deletions (indels) in the target gene, whereas the HDR pathway represents a precise type of genome editing in the presence of a homologous template that enables gene knock-in (KI) (Doudna and Charpentier 2014; Hsu *et al.* 2014). However, the CRISPR/Cas9-mediated KI efficiency is quite inefficient (Li *et al.* 2014; Richardson *et al.* 2016), especially in primary cells (Li *et al.* 2017; Li *et al.* 2018). In addition, the length of some transgenic (TG) cassettes, such as mammary gland bioreactors vectors (> 25 kbp), salivary gland bioreactor carriers (> 20 kbp), and multi-gene co-expression vectors, are very long. Therefore, the precise insertion of a large TG fragment in primary cells remains a huge challenge.

Previous studies have shown that inhibiting the NHEJ pathway or activating the HDR pathway can efficiently promote KI efficiency. However, these pathways mainly focus on immortal cell lines and integrated fragments are also usually short (Chu *et al.* 2015; Maruyama *et al.* 2015; Song *et al.* 2016). Microhomology-mediated end joining (MMEJ) can highly and efficiently integrate large fragments (5.7 kbp to 9.6 kbp) by using 10–50 bp microhomology (Sakuma *et al.* 2015; Sakuma *et al.* 2016; Nakamae *et al.* 2017). However, the targeted integration of large fragments over 20 kbp remains unsuccessful. MMEJ system likely leads to a random integration. In addition, Yoshimi *et al.* showed new single-stranded oligodeoxynucleotides (ssODNs)-mediated KI protocols with CRISPR/Cas9 system that can be applied to any target site in any species and with any donor vector without attaching homology arms (HAs) (Yoshimi *et al.* 2016). However, the high rate of indel mutations at ssODN-mediated conjunction sites can impair the integrity of the inserted transgene.

As an important large animal model in agricultural and biomedical studies, pigs are genetically modified to present desirable traits of economic importance or mimic human diseases (Li *et al.* 2017; Li *et al.* 2018). TG pigs co-expressing three microbial enzymes (β-glucanase, xylanase, and phytase) in the salivary gland have been successfully established (Zhang *et al.* 2018). These enzymes can degrade non-starch polysaccharides and phytate in plants. These TG pigs can significantly promote the digestion of nitrogen and phosphorus in formula feed. Thus, a promising approach that can improve feed efficiency and reduce impacts on the environment is proposed. TG pig lines generated using PiggyBac-mediated transgenesis methods harbor the target gene in the intron of *CEP112*, which encodes a 112-kD centrosomal protein and is localized around spindle poles; however, the structure and function of the target gene remain unclear (Jakobsen *et al.* 2011; Shashikala *et al.* 2013). The pig lines in our study can efficiently express the foreign gene that can be stably inherited by the offspring, which exhibits normal and healthy performance whether it is heterozygote or homozygote. Modified pigs were further established from different pedigrees to breed a new pig variety, which harbored the above-mentioned advantageous traits. The transgene cassette was inserted into the specific genomic locus, including *CEP112* or *ROSA26*, to establish these modified pigs. Therefore, we optimized a CRISPR/Cas9-mediated KI strategy at *ROSA26* and *CEP112* loci in porcine fetal fibroblasts (PFFs) by using different sizes of HAs. Then, we produced a modified pig expressing β-glucanase, xylanase, and phytase in saliva by integrating a transgene fragment (≈ 20 kbp) into the *CEP112* locus.

## Materials and Methods

### Ethics statement

The animal use protocol was in accordance with the Instructive Notions with Respect to Caring for Laboratory Animals issued by the Ministry of Science and Technology of China. The use of animals was approved by the Institutional Animal Care and Use Committee of the South China Agricultural University.

### Plasmid construction

Cas9-gRNA plasmid PX330 was obtained from Addgene (Plasmid #42230). Seven *CEP112* sgRNAs were designed in http://crispr.mit.edu (Supplementary Table S1), and a *ROSA26* sgRNA (R5: 5′-GTGAGAGTTATCTGACCGTA-3′) was used as previously reported (Li *et al.* 2017). The *CEP112* sgRNAs were synthesized, annealed, and cloned into PX330 to form the targeting plasmids PX330-C1–PX330-C7. The transgene was formed by fusing two β-glucanases genes (*bg17A* and *eg1314*), a xylanase gene (*xynB*), and a phytase gene (*appA*) in a head-to-tail tandem array with E2A, P2A, and T2A as linkers between them; the transgene is abbreviated as BgEgXyAp gene (Zhang *et al.* 2018). For the homologous template DNA of the *ROSA26* and *CEP112* loci, the left arm (LA) and right arm (RA) on both sides of the targeted sites (Supplementary Table S2) were amplified and constructed into the upstream and downstream regions of the transgene pPB-mPSP-BgEgXyAp-neoGFP (PB-PSP-BEXA) to form donor vectors.

### Cell culture and transfection

PFFs were isolated from a 30-day old fetus of Duroc pig and cultured in Dulbecco’s modified eagle medium (Thermo Fisher Scientific, Suwanee, GA, USA) supplemented with 10% fetal bovine serum (Thermo Fisher Scientific, Suwanee, GA, USA). Then, the efficiency of induced DSBs was evaluated. PFFs were grown in a culture with up to 80% confluence harvested by 0.25% trypsin/EDTA. Subsequently, 1 × 10^6^ cells per sample were re-suspended in 100 μL of nucleofector solution (Amaxa Biosystems/Lonza, Cologne, Germany), containing 5 μg of CRISPR/Cas9 plasmid and electroporated by the program A-033 using Nucleofector 2b Device (Amaxa Biosystems/Lonza, Cologne, Germany). After the transfected cells were cultured for 2 days, the target region of the cells was amplified with a primer, as shown in Supplementary Table S3; polymerase chain reaction (PCR) products were evaluated by T7 endonuclease I (T7E1) assay as previously described (Li *et al.* 2017). For KI cell line screening, PFFs were co-electroporated with 5 μg of CRISPR/Cas9 plasmid and 10 μg of donor template. After electroporation, cells were distributed into appropriate well plates for subsequent culture and screening.

### Identification of KI cell lines by PCR amplification

One day after co-electroporation with CRISPR/Cas9 plasmid and donor template, cells were transferred into 10 cm plates. Then, G418 (400 μg/mL) selection of the cells was performed for approximately 2 weeks. Fluorescent monoclonal cells were picked and further cultured in 48-well plates. Confluent monoclonal GFP^+^ cell colonies were harvested, from which 4 out of 5 cells were cryopreserved, and the others were lysed for PCR amplification to identify whether the transgene was integrated with specific primers (Supplementary Table S3).

### Generation of cloned pigs by somatic cell nuclear transfer

The positive TG cells were treated with cyclization recombination (Cre) enzyme (Excellgen, Rockville, MD USA) to delete the enhanced green fluorescent protein (EGFP) marker gene before somatic cell nuclear transfer (SCNT). SCNT was performed as previously described (Zhang *et al.* 2018). Approximately 200 reconstructed embryos were surgically transferred to the oviduct of recipient gilt pigs 24 h after estrus was observed. The pregnancy status was detected using an ultrasound scanner approximately 1 month after surgery and monitored monthly until birth. All cloned piglets were naturally born.

### PCR and Southern blot analysis of founder pigs

Genomic DNA from the ear tissue of founder (F0) pigs was isolated according to the protocol of the OMEGA Kit (OMEGA Bio-Tek, Georgia, USA) for PCR and Southern blot analyses. PCR was performed using the primers shown in Supplementary Table S3. The products were resolved by 1% agarose gel electrophoresis and sequenced to determine the occurrence of KI.

For Southern blot analysis, 20 μg of genomic DNA were digested with *Xmn* I or *BsrG* I, separated in a 0.8% agarose gel, and then transferred to a nylon membrane. The membrane was hybridized with digoxigenin-labeled DNA probe, which targets the BgEgXyAp gene. Hybridization and washing were performed according to the procedures of DIG-High Prime DNA Labeling and Detection Starter Kit II (Roche, Mannhein, Germany). The membranes were imaged using the UVP software.

### Gene copy quantification

The PB-PSP-BEXA plasmid was diluted to different concentrations (including DNA copies: 5°, 5^1^, 5^2^, 5^3^, 5^4^, 5^5^) as a standard and was simultaneously subjected to real time PCR with 10 ng of genomic DNA of the KI pigs (using the primers RTQ-copy in Supplementary Table S3). Standard curve was generated according to the copies and CT value of standard DNA, and the copy number of each sample was calculated per standard curve.

### Enzymatic activity assay and Western blot analysis

Saliva was collected from growing KI pigs and age- and breed-matched non-KI pigs. To measure the saliva enzymatic activity of β-glucanase and xylanase in the transgene, 3,5-dinitrosalicylic acid (DNS) method was employed, as previously described (Luo *et al.* 2010; Liu *et al.* 2010; Zhang *et al.* 2018). One unit of β-glucanase activity was defined as the quantity of enzyme that releases reducing sugar at the rate of 1 μmol/min. Vanadium molybdenum yellow spectrophotometry was used to detect phytase activity as described previously by Zhang *et al.* (2018). One unit of phytase activity was defined as the amount of activity that liberates one micromole of phosphate per minute at 39°.

For Western blot analysis, total proteins from saliva were ultrafiltrated using an Amicon Ultra 15 mL centrifugal filter (Millipore, Massachusetts, USA). Then, 20 μg of total protein from saliva were subjected to SDS polyacrylamide gel electrophoresis and transferred onto a polyvinylidene fluoride membrane (Millipore, Massachusetts, USA). The membranes were blocked with 5% non-fat dry milk in tris-buffered saline with Tween-20 (TBST) for 2 hr and incubated overnight at 4° with primary rabbit polyclonal antibodies against BG17A and EAPPA (Zhang *et al.* 2018). The membranes were probed with a salivary amylase antibody (ab34797, Abcam) to confirm equal protein loading. After washing with TBST, the membranes were further incubated with a secondary horse radish peroxidase-conjugated goat anti-rabbit IgG antibody for 2 hr at room temperature and imaged using SuperSignal West Pico-enhanced chemiluminescence kit (Thermo Fisher Scientific, Suwanee, GA, USA). The signals were visualized using the UVP EC3 imaging system.

### Statistical analysis

Data were presented as mean ± SEM and were analyzed using the PASW Statistics 18 (IBM SPSS, Chicago, IL, USA) to assess the statistical significance of their differences. ANOVA was used to assess the differences, and Duncan’s test was employed for multiple comparisons. Differences were considered significant at *P* < 0.05.

### Data Availability

Strains and plasmids are available upon request. The authors affirm that all data necessary for confirming the conclusions of the article are present within the article, figures, and tables. All primers of the article such as gRNA screening, plasmid construction, gene editing cells and pig identification are in Supplementary Table. Supplemental material available at figshare: https://doi.org/10.25387/g3.11341190.

## Results

### Screening of sgRNA with high cleavage efficiency in CEP112 locus

**Figure 1:**
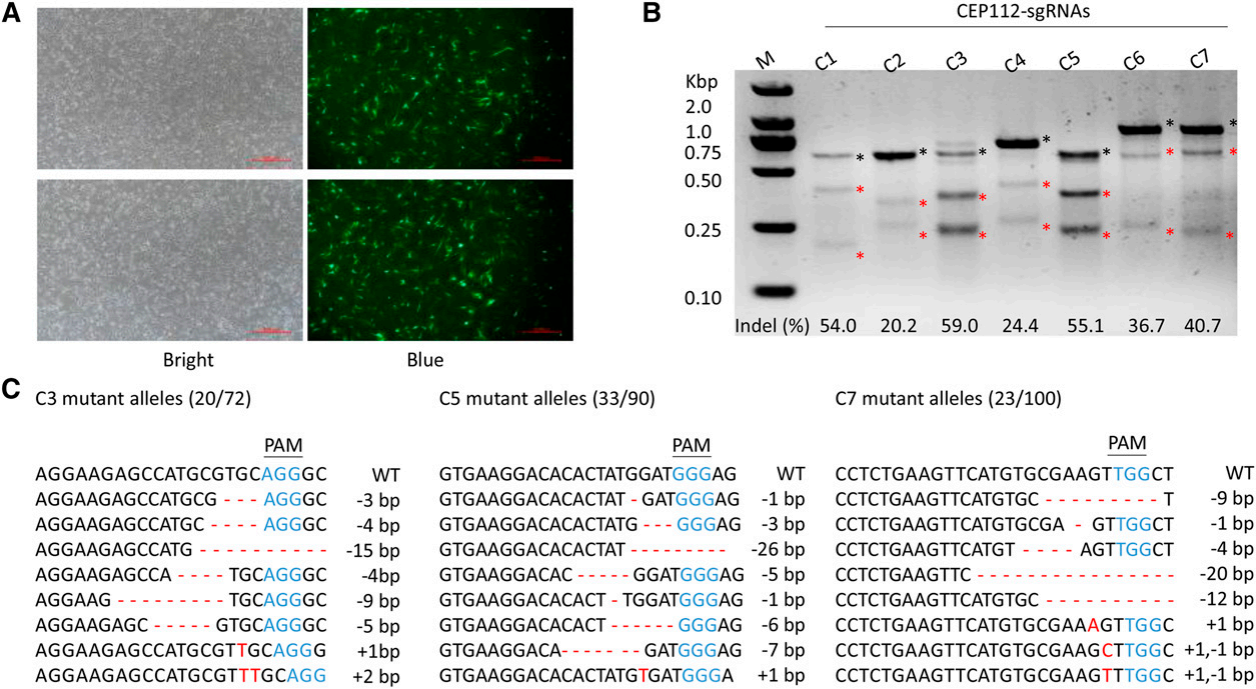
Screening of sgRNA with high cleavage efficiency in *CEP112* locus. (a) Fluorescence expression in PFFs co-transfected with sgRNAs and pBb-ubc-eGFP. (b) Determination of the cleavage efficiency by T7E1 assay. The red and black asterisks indicate cut and uncut bands, respectively. (c) Sanger sequencing further confirmed the cleavage rates of sgRNAs C3, C5, and C7 in the *CEP112* locus. Eight mutant alleles are used as representatives. Indels are shown in red letters and dashes. Protospacer adjacent motif (PAM) sequences are presented in blue.

We designed seven sgRNAs targeting the intron 5 region of the porcine *CEP112* gene. CRISPR-targeting plasmids were transfected into PFFs with a fluorescence expressing plasmid (pBb-ubc-eGFP), which was used as a control to evaluate the transfection efficiency (Figure 1a). The cleavage efficiency of each targeting plasmid was evaluated by a T7EI assay. The results demonstrated that some sgRNAs could efficiently induce targeted mutations. For example, C1, C3, C5, and C7 had more than 40% cleavage efficiency, whereas the others showed a lower efficiency (Figure 1b). C3, C5, and C7 PCR products were cloned into TA vectors and sequenced to further confirm the cleavage rates of the sgRNAs. The percentage of mutant alleles with indels in target sites was 27.8% for C3, 36.7% for C5, and 23.0% for C7 (Figure 1c). We selected C5, the sgRNA with the highest cleavage rate, for subsequent experiments.

### Optimization of homologous templates

**Figure 2:**
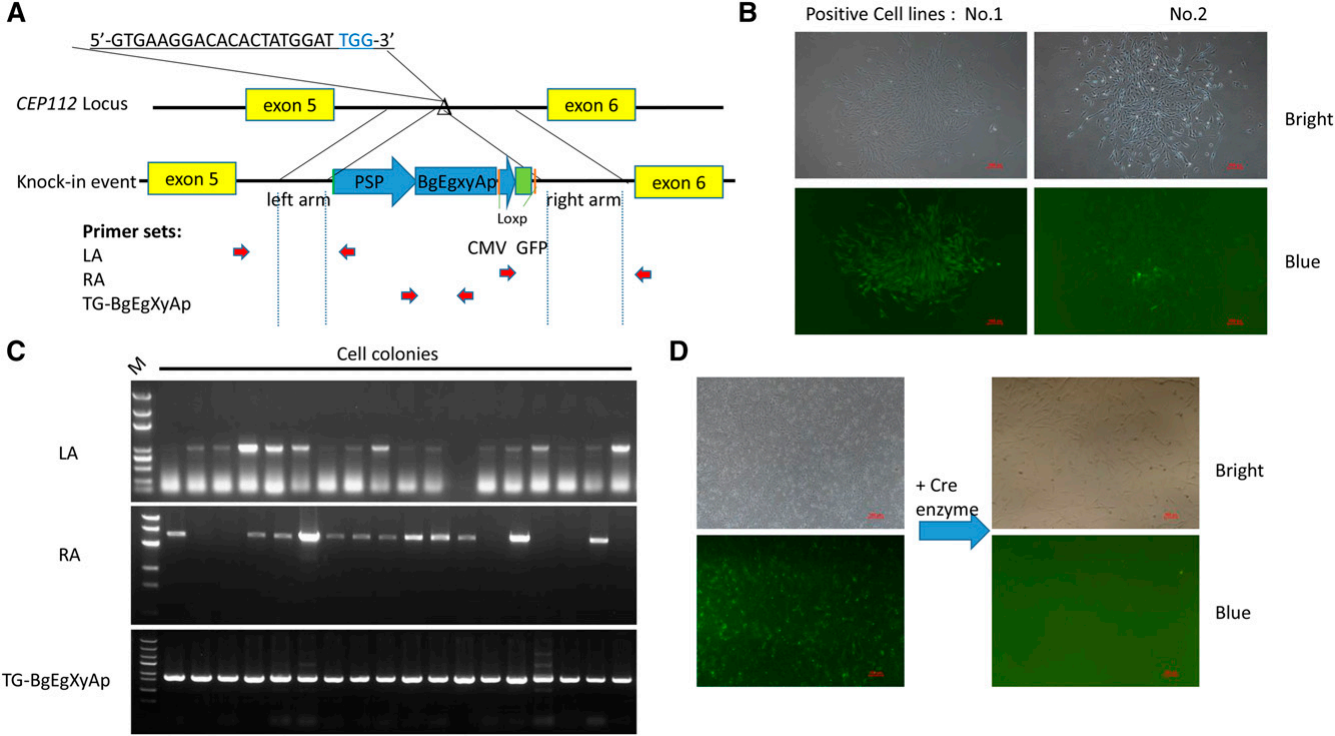
Effects of the length of HA on the screening of KI cell lines. (a) Schematic diagram of the strategy for the insertion of the BgEgXyAp and fluorescence-labeling genes into porcine *CEP112* intron 5. Triangles indicate Cas9 targeting sites, blue letters are PAM sequences, pink dashes indicate loxp sequence, and red arrows are primer sets for PCR assay. (b) Fluorescent monoclonal cell screening. (c) Analysis of KI event by gel electrophoresis. (d) Fluorescent labeling gene was deleted by Cre enzyme before SCNT.

To investigate the effect of length of HAs on the KI efficiency of large fragment (≈ 20 kbp) into porcine genome region, we constructed 6 donor templates of *ROSA26* locus and 4 templates of *CEP112* locus that contain combinations of left and right HAs with different sizes. The primer sets used to amplify HAs for identifying the positive KI cell lines were located at genome and donor vector regions adjacent to HA ends (Figure 2a). Monoclonal cells with EGFP expression (Figure 2b) were picked and genotyped by PCR assay and agarose gel electrophoresis (Figure 2c). The donor plasmid (ROSA26-LA320RA3769) with 320 bp LA and 3769 bp RA had the highest HDR efficiency (7.54% ± 1.28%) in *ROSA26* locus, and the donor plasmid (CEP112-LA340RA3219) with 340 bp LA and 3219 bp RA yielded the highest HDR efficiency (13.61% ± 1.22%) in *CEP112* locus. Generally, a combination of short LA and long RA in donor plasmid has a high KI efficiency. Furthermore, we investigated whether different structures of *CEP112* donor could influence the KI efficiency. We found that circular plasmids had a higher KI efficiency than linearized plasmids when using the donor of 250 bp LA and 3219 bp RA. However, no difference was observed in circular and linear donors when using the donor of 340 bp LA and 3219 bp RA (Table 1).

**Table 1:** Summary of KI events of different proportions of HAs

		Target events			
Plasmid (LARA)*^1^*		First	Second	Third	HDR efficiency (%)
Circular	CEP112-LA1199RA1999	0/7	0/8	0/15	0 ± 0*^a^*
	CEP112-LA2003RA1999	0/13	0/12	0/25	0 ± 0*^a^*
	CEP112-LA250RA3219	2/20	3/22	5/40	12.06 ± 1.08^b^
	CEP112-LA340RA3219	5/38	7/44	14/119	13.61 ± 1.22^b^
	ROSA26-LA320RA1214	0/7	0/9	0/13	0 ± 0*^a^*
	ROSA26-LA320RA2046	0/11	0/10	0/10	0 ± 0*^a^*
	ROSA26-LA320RA3769	1/13	1/19	3/31	7.54 ± 1.28^c^^,d^
	ROSA26-LA1040RA1214	0/6	0/13	0/8	0 ± 0*^a^*
	ROSA26-LA1040RA2046	0/2	1/17	1/19	3.71 ± 1.87^e^
	ROSA26-LA1040RA3769	0/12	0/17	2/43	1.55 ± 1.55*^a^*^*,e*^
Linear	CEP112-LA250RA3219	2/35	1/33	4/64	5.00 ± 1.00^c^^,e^
	CEP112-LA340RA3219	2/18	3/22	4/32	12.42 ± 0.73^b^

1 Donor plasmid, for example CEP112-LA1199RA1999 represents with 1199bp LA and 1999bp RA.

Different letters (a,b,c,d,e) indicates statistical significant differences (*P* < 0.05).

### Generation of BgEgXyAp KI pigs

**Figure 3:**
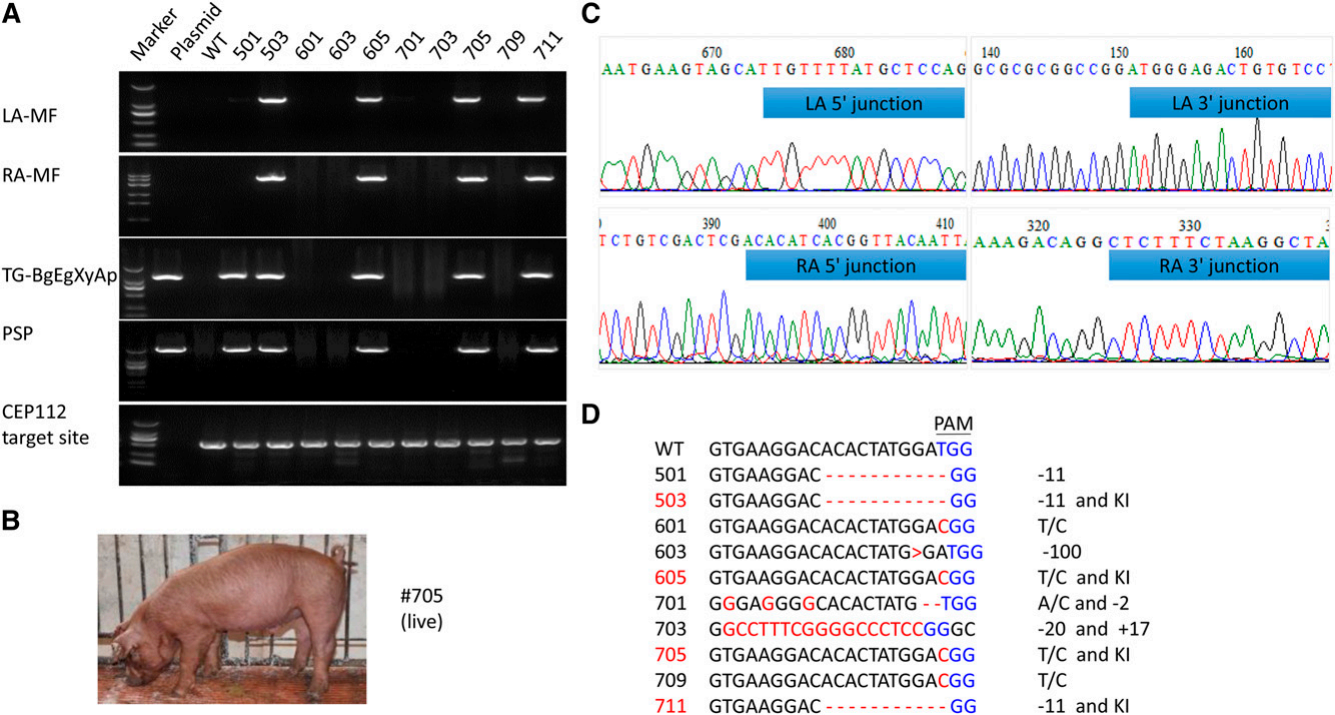
Generation of cloned pigs by using CRISPR/Cas9-mediated HDR system. (a) Genomic DNA of the cloned pigs was amplified by PCR and analyzed by gel electrophoresis. (b) The live KI pig (705) aged 1 month. (c) PCR products of KI loci were sequenced. Blue represents the junction sequences of the HAs and KI locus. (d) Sequences of the targeted *CEP112* loci of all cloned pigs. KI pigs are shown with red numbers. Red letters and dashes represent indels. PAM sequence is displayed in blue.

Three positive KI cell lines that exhibited good morphology and viability were carefully singled out. After fluorescent labeling gene deletion (Figure 2d), cells were pooled and used as nuclear donors to produce modified pigs. We transferred 876 reconstructed embryos into 4 recipient gilts. Two recipients were pregnant and delivered 10 Duroc piglets (Table 2). PCR screening results showed that 4 cloned piglets were heterozygous (503, 605, 705, and 711), thereby achieving a site-specific KI in one allele of the *CEP112* locus (Figure 3a). Among them, only one male piglet (705) lived (Figure 3b). Sequencing results also revealed that the donor was precisely integrated into target without excessive mutations in all KI pigs (Figure 3c). Apart from KI in one allele, all 4 modified pigs contained KO mutations in the other *CEP112* allele. The KO alleles in the 4 pigs were different, including thymine to cytosine mutation (605 and 705) or 11 bp deletion (503 and 711). The six other non-KI pigs showed homozygous KO with 11 bp deletion (501); 100 bp deletion (603) or thymine to cytosine mutation (601 and 709); bi-allelic modification with 17 bp insertion on one allele and 20 bp deletion on the other allele (703); or 2bp deletion on one allele and adenine to cytosine mutation on the other allele (701) (Figure 3d). Taken together, 2 were stillborn (1 KI piglet and 1 non-KI piglet), 5 were born weak and died within 1 month (2 KI piglet and 3 non-KI piglets), and 3 appeared normal at birth and survived to date (1 KI pig and 2 non-KI pigs) (Table 2).

**Table 2:** Embryo transfer data for cloned pigs

Pooled cell lines	Embryos (No.)	Transfers (No.)	Pregnant	Birth		
				Total	KI	Non-KI
3 cell lines	212	1	estrus return	—	—	—
	228	1	normal	4 live piglets	2 live (♂, 1 healthy and 1 dead at day 6)	2 live (♂, 2 dead at day 6)
	218	1	normal	3 live piglets and 3 stillborn	1 live (♂, 1 dead at day 28) and 1 stillborn	2 live (♂, 2 healthy) and 2 stillborn
	218	1	abortions	—	—	—
Total	876	4	2 sows	10 piglets (7 live pigs and 3 stillborn)	4 piglets (1 healty, 1 survive to weaning, and 2 dead)	6 piglets (2 healthy and 4 dead)

### Characterization of gene integration copies and expression patterns in modified pigs

**Figure 4:**
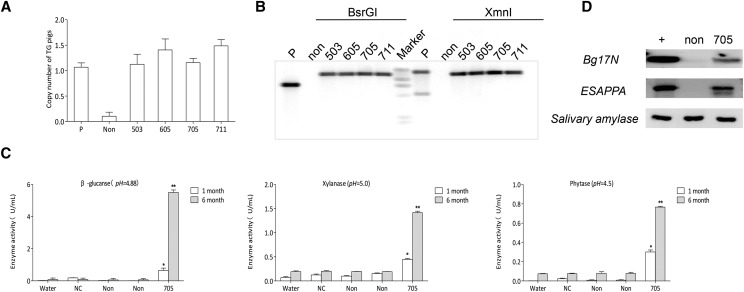
Presence of transgene and expression in KI pigs. (a) Calculation of the copy number of the integrated gene in KI pigs according to the standard curve. (b) Southern blot analysis result of multi-enzyme transgene integration in KI pigs. Genomic DNA of KI pigs was digested by two restriction endonucleases, *BsrG*I and *Xmn*I. (c) Salivary β-glucanase, xylanase, and phytase activity assays of KI pigs at 1 and 6 months. (d) Western blot analysis demonstrated the expression of *Bg17N* and *EAPPA* in the saliva of KI pig (705). Plus sign indicates positive sample; P represents donor vector; NC and Non represent non-modified pig and non-KI pig, respectively. Values are shown as mean ± SEM. The asterisk represents *P* < 0.01.

The donor plasmid was serially diluted to form gradient copies to generate a standard curve and calculate the copy numbers of the transgene with real time PCR. The results showed that all modified pigs have approximately one copy, implying a precise KI without random integration (Figure 4a). Furthermore, Southern blot analysis confirmed that only single copy was inserted into the *CEP112* locus by using two restriction endonucleases *BsrG* I and *Xmn* I (Figure 4b). We collected the saliva from KI and non-KI pigs at 1 and 6 months during the feeding period to detect the enzymatic activities and expression levels of integrated foreign genes. The results showed that KI pig (705) had significant enzyme activity that increased with age. At 6 months, saliva could produce 5.49 U/mL of β-glucanase, 1.42 U/mL of xylanase, and 0.77 U/mL phytase (Figure 4 c). Western blot analysis also confirmed the presence of the protein of β-glucanase (BG17A) and EAPPA (phytase) in the saliva of modified pigs (Figure 4d).

## Discussion

Previous report on gene targeting study showed a correlation between the length of HAs and KI efficiency (0.2–14 kbp) (Thomas and Capecchi 1987). HDR efficiency could be significantly decreased when the length was less than 200 bp. By contrast, the relationship gradually became insignificant when the length reached approximately 14 kbp or more. However, in the presence of artificial nuclease-induced DSBs, KI efficiency can be greatly improved with successful gene editing from HAs lengths ranging from 9 bp to 5 kbp (Orlando *et al.* 2010; Shin *et al.* 2014; Byrne *et al.* 2015). Although numerous studies have exploited engineered nucleases to develop multiple targeting strategies for effective genome editing, KI manipulation remain challenging in various species. In addition, the integration fragments are shorter than < 10 kbp in most reports (Chu *et al.* 2015; Sakuma *et al.* 2015; Sakuma *et al.* 2016; Song *et al.* 2016; Maruyama *et al.* 2015; Nakamae *et al.* 2017). Therefore, precise and efficient genetic modification with large fragment KI (> 10 kbp) must still be further investigated. In this study, we found that a combination of HAs between 300 bp and 3 kbp could yield an efficient KI with efficiency exceeding 10% (*CEP112* locus). Interestingly, shorter and longer HAs could also efficiently enhance CRISPR-mediated integration (Shin *et al.* 2014; Zhang *et al.* 2017). In this study, we observed that circular donor plasmids had a higher KI efficiency than linearized plasmids when using a donor with 250 bp LA and 3219 bp RA; but circular and linear donors had no difference when using a donor with 340 bp LA and 3219 bp RA. We speculated that gRNA can efficiently induce DSBs in donor plasmid after transfection and generate the same structure as linearized plasmid. Meanwhile, we anticipated that different cell lines or DNA loci could affect the outcomes of the HDR repair. Different genetic loci may have different response to the homologous arm lengths, and also the GC content of the homology arms may also affect the efficiency of integration (Shin *et al.* 2014; Zhang *et al.* 2017). In addition, the structure of the donor template might not be essential for the CRIPSR-mediated integration of large fragments. In general, we observed that the use of short and long HAs can achieve a high KI efficiency in PFFs.

Although the donor cells used for SCNT were confirmed positive, only 4 out of 10 cloned pigs were positive for KI. The main reason might be that the selected positive clones were impure and mixed with non-KI cells that could not be distinguished by PCR for KI manipulation. Some mutations shared one allele, but not others such as modified pigs 501, 503, 601, and 605. The difference in genotyping also confirmed that the cell clones were derived from multiple single modified cells. In addition, we observed a high early death rate in the cloned pigs. Abnormal reprogramming of cloned embryos or incomplete embryonic development during pregnancy can account for the early death of cloned animals (Carter *et al.* 2002). Therefore, cloned pigs have high deformity rate and mortality produced by SCNT, and the death of cloned piglets is generally high (58%) before weaning (Liu *et al.* 2015; Schmidt *et al.* 2015). Our results indicated that KI (2 out 4) and non-KI (3 out of 6) pigs had the same mortality rate of 50% that is similar to previous reports (Liu *et al.* 2015; Schmidt *et al.* 2015).

Traditionally, targeted insertions into the *ROSA2*6 or *H11* locus are frequently used for the constitutive or conditional expression of TG pigs (Jakobsen *et al.* 2011; Li *et al.* 2014; Lin *et al.* 2014). In this paper, we established a CRISPR/Cas9-mediated approach for generating KI pigs in the *CEP112* locus. The results revealed that *CEP112* locus supports exogenous gene expression driven by a tissue-specific promoter similar to other safe harbors, such as *ROSA26* (Li *et al.* 2014; Ruan *et al.* 2015; Xie *et al.* 2017). Furthermore, we observed that targeting *CEP112* was more efficient for gene insertion than *ROSA26* for the optimal HAs system The reason might be the chromatin structure of *CEP112*, suggesting that the *CEP11*2 locus may be in an open genomic region and serves as a “hot spot” for gene editing. Many studies have found that KI event likely occurs in regions where DNA can be frequently duplicated or transcribed (Frohman and Martin 1989; Ruan *et al.* 2015).

In summary, we achieved the site-specific targeting of large fragment in *CEP112* locus in pig somatic cells using our optimal HAs system, and established a modified pig model for foreign digestion enzyme expression in saliva, which have the potential to serve as a platform for effective generation of precisely modified pigs for biomedical and agricultural applications.
